# Hepatitis B among University Population: Prevalence, Associated Risk Factors, Knowledge Assessment, and Treatment Management

**DOI:** 10.3390/v14091936

**Published:** 2022-08-31

**Authors:** Syed Ayaz Kazmi, Abdul Rauf, Mohammed Merae Alshahrani, Ahmed Abdullah Al Awadh, Zahoor Iqbal, Raya Soltane, ElSayed Tag-Eldin, Altaf Ahmad, Zulqarnain Ansari, Zia-ur-Rehman

**Affiliations:** 1 Department of Zoology, King Abdullah Campus, University of Azad Jammu and Kashmir, Muzaffarabad 13100, Pakistan; 2 Department of Clinical Laboratory Sciences, Faculty of Applied Medical Sciences, Najran University, Najran 61441, Saudi Arabia; 3 Department of Mathematics, Quaid-i-Azam University, Islamabad 44000, Pakistan; 4 Department of Basic Sciences, Adham University College, Umm Al-Qura University, Makkah 21955, Saudi Arabia; 5 Department of Biology, Faculty of Sciences, Tunis El Manar University, Tunis 1068, Tunisia; 6 Faculty of Engineering and Technology, Future University in Egypt, New Cairo 11835, Egypt; 7 Department of Chemistry, King Abdullah Campus, University of Azad Jammu and Kashmir, Muzaffarabad 13100, Pakistan; 8 RAHMA Islamic Relief, Pakistan, House No. 817, Ammar Chowk, Askari Road Chaklala Scheme III Chaklala Housing Scheme 3, Rawalpindi 46000, Pakistan; 9 Department of Orthopaedics and Spine, Ghurki Trust Teaching Hospital, Lahore 54000, Pakistan

**Keywords:** prevalence of hepatitis B, HBV-associated risk factors, preventive knowledge of HBV, hepatitis B in Azad Jammu and Kashmir, Pakistan, mourning blades, hepatitis B among university students and employees

## Abstract

**Background:** Very few studies have been reported on hepatitis B in the State of Azad Jammu and Kashmir, Pakistan, and none of them are specific to the prevalence and causes of hepatitis B spread among educational institutes. This study aimed to estimate the prevalence of hepatitis B infection and its associated risk factors among the University of AJ and K population. **Methods:** An observational, cross-sectional, and analytical study was conducted with 7015 students and employees. Hepatitis B was detected by rapid immunochromatographic tests (ICTs), enzyme-linked immunosorbent assay (ELISA), and real-time quantitative PCR. A questionnaire and interview method was used to assess the disease knowledge and associated risk factors with hepatitis B through Chi-square, Fisher’s exact test, and paired *t*-test. **Results:** Of the participants, 150 (2.13%) were found positive for the hepatitis B surface antigen (57.3% male and 42.7% female). Only 0.3% participants were found fully vaccinated against the hepatitis B virus. Among ethnic groups, the Syed tribe was found more prevalent for hepatitis B infection (40.6%), while use of contaminated mourning blades (95% CI: *p* = 0.0001) was found as an overlooked risk factor. Hepatitis preventive awareness sessions were found to be very significant (*p* = 0.0001). **Conclusions:** The study showed that an overlooked risk factor is playing a key role in the spread of HBV in a tribe living worldwide, which must be addressed globally to eradicate hepatitis B. In Pakistan, a country-wide annual HBV vaccination program should be launched to control hepatitis B.

## 1. Introduction

Hepatitis B is one of the major global health problems [1,2,3], especially in Asia, Africa, southern Europe, and Latin America [4]. About two billion people are infected with HBV worldwide [2,4,5], and 400 million among them are suffering from chronic HBV infection [6]. HBV mostly spreads through blood and serum, and can also exhibit vertical transmission from mother to child. Unsafe injections, sexual transmission, and vertical transmission are the most common routes of infection for HBV [7,8,9,10,11].

Studies providing a clear picture of hepatitis B among the population of educational institutes are limited. However, in the University of Bangui, 1.3% hepatitis B-prevalent students were found. HBV familial antecedents, sexual activity, and socioeconomic conditions were the main risk factors of HBV infection encountered in the adolescents and young adults [12]. Similarly, a prevalence of 4.7% of hepatitis B among 150 students was found at the University of Uyo, Nigeria. Divorcee students and those with a history of more than five sexual partners were found significantly associated with a high prevalence of hepatitis B [13]. Among Makerere University medical students, an 11.0% hepatitis B prevalence was found, associated with sexual relationships, accidental needle-stick injuries, and unprotected exposure to patients’ body fluids [14]. The prevalence of HBV infection among medical laboratory science students was 6.7% among the participants of the Ghanaian tertiary institution. A total of 43.3% of students were found to be vaccinated against HBV, while sharp-object-based injuries and torn gloves increased the prevalence of HBV infection [15].

Pakistan is highly endemic to HBV with nine million people infected with it [16], but limited data is available to calculate the exact exposure level [17], and there is a steady rise in the infection rate [18]. The reasons may be the lack of proper health facilities, poor economic status, and less public awareness regarding the transmission of major communicable diseases including HBV [6]. Moreover, statistically significant and positive linear correlations between knowledge–attitude (r = 0.536, *p* < 0.05), knowledge–practice (r = 0.022, *p* < 0.05), and attitude–practice (r = 0.026, *p* < 0.05) were observed among Medical College students of Quetta, Pakistan [19]. The last nation-wide survey on hepatitis B was conducted by the Pakistan Medical Research Council from 2007 to 2009 and nearly four million people were estimated to be exposed to the hepatitis B virus, and the actual prevalence of hepatitis B in Pakistan was found to be 2.5%. The vaccine against HBV was introduced in the Pakistan Government’s EPI program in 2009. It has replaced the previous DPT and hepatitis B vaccine, but the data about the country-wide vaccination rate are missing. However, in 2021, Soomar et al. [20] conducted a study and reported that two-thirds of the health care workers were found completely vaccinated in secondary care hospitals in Sindh, Pakistan. Similarly, a study was conducted on the prevalence of HBV and HBV vaccination coverage among health care workers of the tertiary hospitals of Peshawar, Pakistan and reported that the highest coverage was found among doctors, followed by administrative staff, general and assistant staff, technicians, and nurses. Overall, 73.4% of participants were found to be fully vaccinated against the hepatitis B virus [21].

Due to a lack of studies, no sufficient data are present regarding the prevalence of hepatitis B, hepatitis B virus vaccination status, and associated risk factors among students to generate a hepatitis control policy for educational institutes at the national level. Most of the previous studies targeted different small groups, especially without involving those who have no study concern with disease knowledge and severity. Therefore, these studies do not accurately reflect the overall prevalence of hepatitis B infection among the population of educational institutes in Pakistan, more specifically in AJ and K. This study aimed to estimate the hepatitis B prevalence, knowledge and associated risk factors assessment, and management of hepatitis B treatment among the University of AJ and K population.

## 2. Materials and Methods

### 2.1. Population and Blood Sampling

Students and employees from the University of Azad Jammu and Kashmir, Muzaffarabad were included for the observational, cross-sectional, and analytical study. The study was approved by the Board of Advanced Studies and Research (BASR) in the University of Azad Jammu and Kashmir, Muzaffarabad as approval letter number F-BASR/(41st M)/16-40/1669-71/2018 and it took almost 3 years to complete. In this observational, cross-sectional, and analytical study, the sample size was calculated using Raosoft software online sample size calculator owned by Raosoft Inc., 6645 NE Windermere Rd, Seattle, WA 98115, USA (http://www.raosoft.com/samplesize.html accessed on 15 March 2018). The population size of the AJ and K state was reported to be 4.045 million after the 2017 census. The response distribution was set at 50%, while the confidence interval was set at 95% and the error margin at 5%. The minimum recommended sample size after analysis was found to be 385. However, 7015 individuals were found willing to participate in the study. Demographic data including age, gender, ethnic background, and previous medical history were also obtained from all participants. Five milliliters of blood were drawn from each individual and, after centrifugation, the serums were separated for storage at −20 °C until use.

### 2.2. Laboratory Testing

All serum samples were tested for the presence of the hepatitis B surface antigen (HBsAg) using immunochromatographic test (ICT) devices and then confirmed through an enzyme-linked immunosorbent assay (ELISA) according to the manufacturer’s instructions (CTK Biotech, San Diego County, CA, USA).

### 2.3. HBV DNA Extraction and Amplification

The viral DNA was extracted from the blood samples of HBsAg-positive individuals via the remolded DNAzol method optimized by Rauf et al. [22]. The extracted DNA from the serum of HBsAg-positive people was utilized for the PCR amplification of HBV surface quality. Because of the low concentration of the viral DNA in the serum, the HBV DNA was amplified in two rounds of PCR.

For the first round of PCR, following primers were used:

Forward: 50-CATCCTGCTGCTATGCCTCATCT-30
Reverse: 50-CGAACCACTGAACAAATGGCACT-30

For the nested PCR (i.e., second round of PCR) the primers used were as follows:

Forward: 50-GGTATGTTGCCCGTTTGTCCTCT-30
Reverse: 50-GGCACTAGTAAACTGAGCCA-30

### 2.4. Liver Function Tests (LFTs) and International Normalized Ratio (INR)

LFTs of the HBV-positive individuals were performed by using a Merck Microlab-300 analyzer (ELITech-Group, Puteaux, France) for the detection of bilirubin, ALT, and alkaline phosphate level. All the procedures were adopted according to the Randox Laboratories Clinical Chemistry Reagents, LFT Kit. The INR of the patient was calculated by using a standard INR-calculating formula in which the ISI value of 1.4 was taken as given by the Merck Company for its thromboplastin reagent.

### 2.5. Detection of HBeAg

The hepatitis B envelope antigen (HBeAg) as a clinical marker for the detection of the active replication of HBV and high blood infectivity was also tested through HBeAg immunochromatohraphic test devices (SD BIOLINE HBeAg ICT devices, Abbott Laboratories(Pakistan) Limited, Karachi, Pakistan).

### 2.6. Risk Factors and Knowledge Assessment

A comprehensive questionnaire for knowledge assessment previously used by Noman ul Haq et al. [19] was filled by each candidate before and after awareness sessions. Awareness was established through a multimedia presentation and the distribution of awareness material. Risk factors were assessed through the fulfilment of a suitable questionnaire by each participant, which was especially designed for the university students and employees.

### 2.7. Statistical Analysis

For the present study, GraphPad Prism Version 7.04 (GraphPad Software, San Diego, CA, USA) was used to analyze the data. The relationship between disease and associative risk factors was assessed through a Chi-square test (at *p* = 0.05 with 95% CI) and again through a Fisher’s exact test (at *p* = 0.05 with 95% CI) to assess valid outcomes. Similarly, for knowledge assessment, the calculation was performed two times, including before awareness sessions as well as after awareness sessions. For this purpose, the Chi-square test, Fisher’s exact test, and *t*-test were used to maintain the validity of the results at *p* = 0.05 with 95% CI. A *p*-value of 0.05 or less with a 95% confidence interval was used as the cut off value for calculating statistical significance. To find out the relationship between sex and HBsAg prevalence, a two-tailed Chi-square test (at *p* = 0.05 with 95% CI) was used. With a 95% confidence interval, the ranges of relative risk (Koopman asymptotic score), odds ratio (Baptista–Pike), sensitivity (Wilson–Brown test), and specificity (Wilson–Brown test) were calculated.

### 2.8. Treatment Management for Hepatitis B

Treatment of the HBV-positive patients was arranged with the help of the Hepatitis Control Program, Government of Azad Jammu and Kashmir, Pakistan. Quantitative real-time PCR was used to evaluate the serum viral load of the participants after each 3 month treatment period until it was found negative. Similarly, the liver function tests (LFTs) were also observed after intervals. The process was repeated two times even after the detection of zero viral load to confirm the HBV DNA negativity in the serum. Outcomes of the treatment were observed to conclude the treatment efficacy.

## 3. Results

### 3.1. Overall Prevalence of Hepatitis B

**Figure 1:**
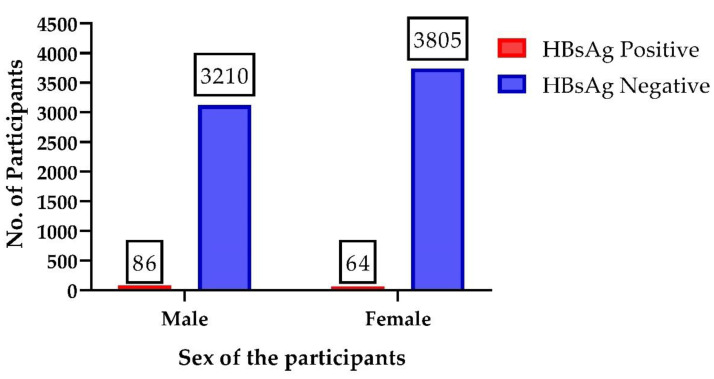
This figure shows the prevalence of hepatitis B in the studied population (2.31%) in which the percentage of hepatitis B surface antigen (HBsAg) was higher in the male (57.3%) population as compared to the female (42.7%) population.

The overall prevalence of hepatitis B with associated detection markers is shown in Table 1. A total of 7015 individuals were screened for hepatitis B surface antigen (HBsAg) by using ICT devices. A total of 150 (2.13%) individuals were found positive for HBsAg in the initial screening. Out of these 150 individuals, 86 (57.3%) were males and 64 (42.7%) were females, as shown in Figure 1. This relationship of sex was found associated (*p* = 0.0040) with HBsAg prevalence. The relative risk, odds ratio, sensitivity, and specificity were found to be 1.158 to 2.191, 1.158 to 2.242, 0.4933 to 0.6497, and 0.5331 to 0.5567, respectively with 95% CI. All of the 150 ICT-positive individuals were further subjected to ELISA for quantitative confirmation of HBsAg and were found with the same results. Of the 150 ELISA-positive individuals, 39 (26%) individuals (33 male and 6 female) were found positive for the presence of HBV DNA through qPCR. The hepatitis B envelope antigen (HBeAg) as a clinical marker for the detection of active replication of HBV and high blood infectivity was also tested through HBeAg immunochromatohraphic test devices (HBeAg ICT). Only 2 (5.1%) individuals (1 male and 1 female) were found positive for the presence of HBeAg out of 39 HBV-infected individuals, showing that the virus was in an active state in the liver of those 2 individuals.

### 3.2. Age-Wise Prevalence of Hepatitis B

In the present study, the total population size was 7015, the mean age with 95% confidence interval (CI) was 23.6 ± 0.17 years, the median age was 22 years, and the mode of age was found to be 21 years of age. Four age groups were used to describe the results regarding to the age of population. These age groups were 16–30, 31–45, 46–60, and above 60 years of age. A total of 6270 (89.4%) individuals were found in the age group between 16 and 30 years, and from them, 138 (2.2%) individuals were found positive for HBsAg. Similarly, the age group between 31 and 45 years included 504 (7.2%) individuals, with no individual found positive for HBsAg. The highest presence of HBsAg was found in the age group between 46 and 60 years. Of 228 (3.2%) individuals screened in this age group range, 12 (5.3%) individuals were found positive for HBsAg. The last age group, which included above 60 years individuals, had no individual found positive for the presence of HBsAg, i.e., 13 (0.2%) out of the total individuals. Table 2 shows all the results regarding age-wise prevalence of hepatitis B.

### 3.3. Prevalence of Hepatitis B Regarding Social Status of the Participants

The screened population comprised two distinct statuses including students and employees. Table 3 shows the prevalence of hepatitis B among students and employees of the University of AJ and K, Muzaffarabad. Of the screened population, 9.5% of the employees of the University were found positive for the presence of HBsAg as compared to the low prevalence rate of 1.6% in students of the University of Azad Jammu and Kashmir, Muzaffarabad, Pakistan. The high prevalence of HBsAg among employees of university might be due to their long exposure duration with possible risk factors during their daily routine.

### 3.4. Viral Load of the PCR-Positive Individuals

The HBV viral load of the positive individuals was detected through real-Time PCR (RT-Quantitative PCR), as shown in Table 4. This was conducted owing to its importance for the treatment against HBV. Normally, the patients with a viral load below 10,000 IU/mL are included in the group that does not require any treatment at that early stage. No such case was found during the present study. The HBV viral load above 10,000 IU/mL is said to be the risky range of virus which could lead to liver damage. We divided this group into two sub-groups: the first one had those with a viral load between 10,000–20,000 IU/mL while the second included those with a viral load above 20,000 IU/mL. Only nine (23.1%) individuals were found in the group with viral load from 10,000–20,000 IU/mL. On the contrary, the remaining 30 (76.9%) HBV-positive individuals were found in the high-risk group with a viral load above 20,000 IU/mL.

### 3.5. Liver Function Tests (LFTs) of the HBV-Positive Individuals

The results of LFTs are shown in Table 5. LFTs of the positive HBV individuals were performed by using a Merck Microlab-300 analyzer for the detection of bilirubin, alanine transaminase level, and alkaline phosphate level. Our results found 18 (46.2%) individuals within the normal range of LFTs while the remaining 21 (53.8%) were found possessing abnormal ranges of bilirubin, ALT, and ALP levels, indicating liver damage due to HBV infection.

### 3.6. Prothrombin Time Test (PT-Test) or International Normalized Ratio (INR) (n = 39)

The INR of the patient was calculated by using a standard INR-calculating formula given by the Merck Company and the results are shown in Table 6. Only 3 (7.7%) patients were found to have higher PT or INR range. The patients were suffering from oral bleeding problems due to hypoprothrombinemia, which might be the result of liver damage due to HBV. The remaining 36 (92.3%) individuals were found in the normal range of prothrombin time.

### 3.7. Risk Factor Assessment Associated with Hepatitis B Spread among Studied Population

Risk factors were assessed through obtaining a filled questionnaire from each participant, which was especially designed for the university students and employees. A two-tailed Chi-square test was calculated using GraphPad Prism Version 7.04 to find out the relationship between hepatitis B and associative risk factors. A *p*-value of 0.05 or less was used as the cut-off value for calculating statistical significance. Table 7 shows the relationship of dental treatment (*p* = 0.0008), history of hospitalization (*p* = 0.018), other immunosuppressive diseases (*p* = 0.0002), tattooing/piercing on any part of the body (*p* > 0.0001), and use of contaminated mourning blades (*p* < 0.0001) with hepatitis B was found significant through a Chi-square test and was confirmed significant through a Fisher’s exact test. The use of contaminated mourning blades was only found in 1 of the 13 tribes of the participants, called the Syed tribe.

### 3.8. Knowledge Assessment of the Participants before and after Awareness

A comprehensive questionnaire for knowledge assessment was filled by each candidate before and after awareness sessions. Awareness was established through a multimedia presentation and the distribution of awareness material. Table 8 shows that the percentage of right answers before awareness was 32%, which was too low, while after awareness this percentage rose to 88.5%. To calculate the significance of awareness sessions on the knowledge of participants, we used a Chi-square test (*p* = 0.05, CI = 95% and df = 1), Fisher’s exact test (*p* = 0.05 and CI = 95%), and paired *t*-test at *p*-value = 0.05. From all three statistical tests, a significant positive correlation between awareness sessions and knowledge level was found.

### 3.9. Prevalence of Hepatitis B among Different Tribes

In the present study, the population size was 7015, which was composed of the participants from 13 tribes. Among tribes, 61 (7.0%) individuals were found HBsAg-positive out of 871 total individuals in the Syed tribe, which was highest among all tribes, as shown in Table 9, and the sharing of contaminated mourning blades was assessed as an overlooked risk factor behind the spread of hepatitis B in the Syed tribe, as shown in Table 7. Similarly, the second-highest prevalence was found in the Abbasi tribe, where we found 10 (2.4%) HBsAg-positive individuals out of 421 total individuals, and in the Khawaja tribe we found 22 (2.4%) individuals positive for HBsAg out of 936 total individuals. According to the prevalence rate of HBsAg, the Awan tribe was found to have the third-highest prevalence among all other tribes. A total of 18 (2.3%) were found positive for HBsAg in the Awan tribe out of 788 total individuals. We also found 1.6% HBsAg prevalence in the Qureshi tribe, 1.3% in the Rajpoot tribe, 0.9% in both the Sudhan and Mughal tribes, 0.7% in the Chaudhary tribe, and 0.5% in the Sheikh tribe, and found no prevalence in the Balti, Pakhtoon, and Peerzada tribes.

### 3.10. Previous Medical History of the Participants

Data regarding previous medical history were obtained from each participant, as shown in Table 10. HBV-vaccinated individuals were found to be 0.3% of the total participants. A total of 0.8% felt sick with hepatitis A, 1.4% had a history of jaundice, 2.4% were hypocalcemic, 9.4% faced tuberculosis, 37.5% had dental problems and visited dental clinics, 0.03% individuals had a transplantation history, gastric ulcer was found in 0.6% individuals, 0.2% individuals were found with gall bladder surgery, 6.2% individuals had a typhoid history, 0.6% had chicken pox in their life, 1.2% were found with vision abnormalities, 0.2% were found with renal stones, 2.3% were found with a history of appendicitis, 3.7% told us that they were facing allergy issues, 0.07% individuals had a history of cardiac problems (all were male), 0.09% had faced pneumonia, and 0.04% individuals told us that they felt sick with measles.

### 3.11. Treatment Management for the Acute and Chronic HBV-Postive Participants

As mentioned earlier, the present study was an observational, cross-sectional, and analytical study, and it was focused mainly on prevalence assessments. However, the study also aimed to allocate the treatment facilities for the hepatitis B-positive participants free of cost. For this, the treatment of the HBV-positive patients was arranged with the help of the Hepatitis Control Program of the Government of Azad Jammu and Kashmir (https://health.ajk.gov.pk/hepatitis-control-program accessed on 2 August 2022), Pakistan. Quantitative real-time PCR was used to evaluate the serum viral load of the participants after each 3 month treatment period until it was found to be negative. Similarly, the liver function tests (LFTs) were also observed after intervals. The process was repeated two times, even after the detection of zero viral load to confirm the HBV DNA negativity in the serum. Outcomes of the treatment were observed to conclude the treatment efficacy.

According to the government hepatitis control policy and treatment facilities, Entecavir tablets (0.5 mg and 1 mg) were suggested to patients by a physician initially as an antiviral drug. Moreover, for liver regeneration and for proper digestive functioning, Silliver (Silymarin 200 mg) and Liv-52 Syrups (herbal–mineral concoction with hepatoprotective effects) were given. Of the 39 HBV patients, with the help of the above-mentioned prescription, 37 (94.9%) patients with acute HBV infections were found with good health and totally reduced viral loads within the first 6 months of the treatment period. Quantitative real-time PCR was used to evaluate the serum viral load of the participants after each 3 month treatment period until it was found negative. Similarly, liver function tests (LFTs) and complete blood count (CBC) were also observed after intervals. The process was repeated two times, even after the detection of zero viral load to confirm the HBV DNA negativity in the serum. However, the remaining two (5.1%) participants (HBeAg-positive) were found to have an increase in the viral load and in the ALT level. Those participants were subjected to the chronic HBV infection treatment with pegylated interferon therapy.

## 4. Discussion

HBsAg greater than 7% in the adult population was reported by Hodges et al. [23] and it was included in high endemicity for HBV infection. A similar study about the prevalence of hepatitis B virus (HBV) infection among newly admitted students of the University of Jos, Jos Plateau State Nigeria was conducted by Odinachi et al. [24] and they reported 16.67% of chronic hepatitis B infection in students of the University of Jos. Both of these studies showed high prevalence of hepatitis B as compared to the 2.13% prevalence of HBsAg in the University of Azad Jammu and Kashmir, Muzaffarabad.

HBV infection by gender distribution showed that males had (11.33%) a prevalence higher than their female counterparts with 5.33% [24]. The study of Odinachi et al. [24] showed the same relation between male and female HBV infection prevalence rates as we found in the present study. Out of 150 HBsAg-positive individuals, 86 (57.3%) were males and 64 (42.7%) were females. The same rate of high prevalence of hepatitis B among males in both studies clearly indicates a high exposure of males with risk factors as compared to females. In the current study, the tattooing on body parts, injuries, and cuts through the use of ritual mourning blades were only found in male participants.

When considered the percentage prevalence based on age distribution, the present study showed a higher prevalence rate of HBV among the age group 25–29 (28.57%). The reason may be due to high sexual transmission among members of the age group [25]. On the contrary, [25] transmission of HBV in this respective population differs from the study population of University of Azad Jammu and Kashmir, Muzaffarabad. We did not ask any question about the free sexual activities of the participants in the present study and we found a high prevalence of HBV infection in age group 45–60, which was 5.3% (12/228). The sharing of personal belongings, visit to beauty parlor/barber shop and a history of injections were assessed in 100% participants. A total of 62.5% were found positive due to tattooing/piercing on different parts of their body.

Li et al. [26] found a 9.3% prevalence of hepatitis B in the age group of 60–69 years, but we found not even a single hepatitis B-positive individual in the age group for above 60 years individuals. There is a difference between both studies regarding the prevalence rate of hepatitis B in different age groups. Similarly, both these studies differ on the basis of risk factors among the studied populations. Male gender, a history of surgical operations, at least one HBsAg-positive family member, non-vaccination for 15–59-year-old adults and a blood transfusion history for adults above 60 years of age were marked as potential risk factors in the general population of the Anhui Province, China. In the present study, we found a history of blood transfusion in 16.7% whereas sharing of personal belongings, visit to beauty parlor/barber shop, and a history of injections were assessed in 100% of participants.

HBV is responsible of 49.6% of acute viral hepatitis cases in Turkey. Turkey is considered as a medium endemicity (2–7%) region of HBV infection [27]. The percentage of hepatitis B virus infection in the general population of Pakistan was found to be 4.3318% ± 1.644% in another study [28]. The prevalence estimates (approximately 0.4%) for the general population have underestimated the true burden of chronic HBV infection and recent data indicate that the migration of people with an existing HBV infection has had an important impact on the prevalence of the HBV infection in the United States [29]. As compared to all these studies among different countries of the world, the present study showed 2.13% of hepatitis B prevalence in the State of Azad Jammu and Kashmir, Pakistan. In the present study, the percentage prevalence (2.13%) of hepatitis B was higher than the 0.4% prevalence of hepatitis B in the United States; lower than the 4.3% prevalence of hepatitis B in the general population of Pakistan and ranging from 2–7% accepted endemicity of hepatitis B in Turkey. This might be due to the different population selection criteria and exposure to different risk factors of all these studies.

The prevalence of hepatitis B among dentists was reported to be 10.8% in Brazil, 9% in USA, and 7% in Germany [30]. In the study by Nagao et al. [29] the mentioned populations were dentist communities of Brazil, USA and Germany, and dentists are always included in possible risk factors of hepatitis B spreading. So, it is not sure that the dentist community practice for themselves with those surgical instruments which they use for their patients/customers. However, the percentages of prevalence mentioned by Nagao et al. [30] were much higher than the 2.31% prevalence of hepatitis B in the population found in the current study. This indicates that poor practice and non-hygienic conditions adopted by dentists in Brazil, USA, and Germany were the main reasons of such a high prevalence of hepatitis B among them.

In a similar study in China, the overall prevalence of HBsAg was found to be 7.44% (out of 8895 total participants) and the prevalence of HBsAg was significantly higher for males (8.2%) when compared with females (6.8%). Prevalence was lowest among children less than 5 years old (0.3%) and highest among those aged 60–69 years (9.3%). Among adults 15–59 years old, the risk factors were male gender, a history of surgical operations, at least one HBsAg-positive family member, and non-vaccination. For adults older than 59 years, the risk factor was a blood transfusion history [26]. The study of Li et al. [26] showed enrolment of 8895 participants out of which they found a 7.44% prevalence of hepatitis B, but in the present study we enrolled 7015 participants and we found a 2.13% prevalence of hepatitis B. This difference is directing us to focus on the population demography related to education level. We found a lower prevalence of hepatitis B as compared to Li et al. [26] because our studied population belongs to the highly educated community of the State of Azad Jammu and Kashmir, Pakistan. However, both studies showed higher prevalences of hepatitis B in males as compared to females.

Another study conducted by Ishida et al. [31] focused on the prevalence of hepatitis B in the rural ethnic populations of Northern Thailand. The positivity rate of HBV and HCV infection in each tribe ranged 4.7% (Akha)–22.6% (Lahu) and 2.0% (Hmong and Akha)–8.1% (Shan), respectively. The prevalence of HBV was significantly different (*p* = 0.005) among the tribes as compared to HCV [31]. They found 22.6% HBsAg positivity in the Lahu tribe which was the highest among all tribes, in contrast to the 4.7% HBsAg positivity in the Akha tribe, which was the lowest among all tribes. They found just 658 positive individuals from seven ethnic groups: Lahu, Lisu, Shan, Red Karen, White Karen, and Hmong in the Mae Hong Sorn Province and Akha in the Chiang Rai Province. In comparison to this study, we explored 13 different tribes in the present study with 7015 total population size and found the Syed tribe with the highest prevalence of hepatitis B with 61 (7.0%) positive individuals for HBsAg out of 871 total individuals. This highest prevalence of hepatitis in the Syed tribe might be due to the religious practice of mourning and sharing mourning equipment. In Muzaffarabad, Azad Kashmir, most of the population of the Syed tribe belongs to the Shiite belief who use mourning blades during their religious practice, which may serve as the potential risk factor behind the spread of hepatitis B in this tribe. In our study, three different tribes did not possess any single individual positive for HBsAg.

The main sources of knowledge regarding hepatitis B were the media and medical staff. The mean knowledge score was 14.8 ± 4.9; 76.7% of the respondents had scores >50%. Particular gaps were detected relating to knowledge of unprotected sexual intercourse; 45.6% patients were not aware of the potential asymptomatic course of HBV infection and 41.2% were not aware about chronic HB treatment. A patient’s low educational level was negatively associated with a high knowledge level [32]. The study conducted by Ganczak et al. [32] in Poland was objectively similar to our study but they selected the patient population of primary care clinics for this purpose and this created a difference of results between both studies. A total of 87.3% positive response about dental surgery in their study is higher than 37.5% of the same question asked in our study during risk factor assessment. Similarly, 86.6% positive response about tattooing in their study is also higher than 62.5% of the same question asked in our study during risk factor assessment. A total of 90.2% positive response about the knowledge related to availability of an efficient hepatitis B vaccine in their study is approximately 2.5 times higher than the 36.6% positive response against the same question in our study. Another main difference between both studies is the conduction of awareness sessions with post-session knowledge assessment to find the effectiveness of the awareness session, and we found they raised the percentile of awareness about hepatitis B from 32% to 88.5% with respect to right answers to the questions.

Of 322 distributed questionnaires, 322 were returned with a response rate of 100.0%. The majority of the students (91%) were of the age group 20–24, of which 232 (72%) of the respondents were males. The majority (95.3%) of students were not fully vaccinated against hepatitis B and 48.4% of the students were not aware of the availability of post-exposure prophylaxis for HB. Mean scores for knowledge and practice were 11.52 ± 2.37 and 2.76 ± 1.1, respectively [33]. The study conducted by Mesfin and Kibret [33] in the Haramaya University, Ethiopia was focused only on medical and health science students. Students of medical and health sciences always have direct knowledge of infectious disease such as hepatitis B as they study these diseases as compulsory topics. The population size of the study was just 322 individuals, which is approximately 21.8 times smaller than the 7015 population size of our study, and our study focused all the students of the university, including both science as well as arts students. So, we cannot compare both studies due to significant differences in diseases awareness between them.

A study was conducted by Pido and Kagimu [14] among Makerere University medical students and reported that over 90% of the students had no history of prior hepatitis B immunization and 11.0% hepatitis B surface antigen prevalence was found among them. Similarly, Sannathimmappa et al. [34] conducted a study among preclinical-year medical students and reported that the majority of students did not know their vaccination status, whereas only 23.5% of the students were fully immunized. In comparison, the current study was conducted among the students and employees of the University of Azad Jammu and Kashmir, Pakistan, and 2.13% hepatitis B surface antigen prevalence was found, while 0.3% (including 14 male and 7 female) participants were found with complete hepatitis HBV vaccination. The difference between the vaccination statuses of the studies is due to different study population backgrounds as well as the population sizes.

A cross-sectional study was conducted at eight medical universities in Vietnam and reported that, among 2000 participants, 84.2% were found already tested for hepatitis B and 83.9% were found vaccinated against HBV [35]. However, in Azad Jammu and Kashmir, we found only 0.3% participants vaccinated against HBV and none of the participants was found already tested for hepatitis B. This difference is due to the population backgrounds of these two studies. Nguyen et al. [35] conducted their study among the students of medicines in Vietnam. Medical students are familiar with most of the diseases such as hepatitis B and their preventions. In contrast, in the current study, we assessed hepatitis B and its vaccination status among participants of various disciplines. A total of 63.4% of participants even did not hear about hepatitis B in their life. So, the difference between these two studies is due to the difference of awareness level between the studied populations.

## 5. Conclusions

In the present cross-sectional study among the university population, it was found that 2.13% of hepatitis B infections were significantly associated with dental surgery, history of hospitalization, other immunosuppressive diseases, and tattooing/piercing on any part of the body. The vaccination status of the studied population was found to be very low (0.3%) and no correlation was found between hepatitis B prevalence and HBV vaccination. Moreover, it was found that the knowledge of the participants about hepatitis B was low (nearly 32%) and it was raised up to 85% by the conduction of disease awareness sessions by the authors. Additionally, this study specifically indicated that the use of contaminated mourning blades for the ritual of Zanjeer Zani was found as an overlooked risk factor among participants of the Syed tribe. While dealing with the management of the treatment of HBV infected participants, it was observed that the Entecavir drug was found effective in the case of acute HBV infection, whereas PEGylated interferon therapy was found effective in chronic HBV infection cases.

This study suggests that the Government of the State of AJ and K, Pakistan should take steps to develop a policy for all educational institutes for the proper screening and related assessments so that comprehensive data can be generated to understand as well as to control infectious diseases such as hepatitis B at a national level. This study also recommends that the HBV vaccination should be make compulsory, especially for educational institutes.

## Figures and Tables

**Table 1:** This table shows the prevalence of HBsAg, HBV, and HBeAg among the participants.

Sex	Total Participants	HBsAg Positive	HBsAg Negative	HBV Positive	HBV Negative	HBeAg Positive	HBeAg Negative
**Male**	3210	86	3124	32	54	1	31
**Female**	3805	64	3786	7	57	1	6

**Table 2:** Prevalence of hepatitis B in different age groups is shown in the table.

Age Groups	No. of Individuals	Standard Deviation	Median Age	Mode of Age	Mean Age with 95% (Z* = 1.96) Confidence Interval (CI)	HBsAg-Positive Individuals
16–30	6270	3.3	21	21	21.5 ± 0.08	138 (2.2%)
31–45	504	13.9	36	35	36.8 ± 1.2	0
46–60	228	27.9	50	50	51.4 ± 3.6	12 (5.3%)
Above 60	13	40.1	63	63	63.6 ± 21.8	0
Total Population	7015	7.2	22	21	23.7 ± 0.17	150 (2.13)

Z* was the standard score used for the calculation of probability during data analysis.

**Table 3:** This table shows hepatitis B prevalence regarding social status of the participants.

Population Distribution	No. of Individuals	Prevalence of HBsAg	Percentage
Employees	493	47	9.5%
Students	6522	103	1.6%
Total	7015	150	2.13%

**Table 4:** In this table, viral load of the HBV-positive (*n* = 39) individuals is shown, distributed in three categories.

Viral Load	No. of Individuals	Percentage
<10,000 IU/mL	0	0%
10,000–20,000 IU/mL	9	23.1%
>20,000 IU/mL	30	76.9%

**Table 5:** In this table, bilirubin, ALT, and ALP levels in the serum of HBV-positive individuals discriminate between healthy and damaged liver patients.

Test	Normal Range	No. of Individuals within Normal Range	%	No. of Individuals above Normal Range	%
Bilirubin	0.2–1.0 mg/dL	18	46.2	21	53.8
ALT	Male 0–43 U/L				
	Female 0–36 U/L				
ALP	Adult 80–306 U/L				
	Child up to 645 U/L				

**Table 6:** The table shows the prothrombin time test for the PCR-positive individuals (*n* = 39), which was calculated under ISI value 1.4 Merck Company for its reagents.

Prothrombin Time	INR = (PT Patient/PT Normal)^ISI^	Indications	No. of Individuals (*n* = 17)	%
**Below 11 s**	<0.8	Hyperprothrombinemia	0	0
**11 to 13.5 s**	0.8 to 1.1	Normal	36	92.3
**Above 13.5 s**	>1.1	Hypoprothrombinemia	03	7.7

**Table 7:** Assessment of associated risk factors with hepatitis B spread among the studied population.

Factors	Responses	Overall Responses	Frequencies Diseased	Frequencies Non-Diseased	*p*-Value (0.05)	Statistical Significance
**Jaundice/hepatitis history**	Yes	100 (1.4%)	02	98	0.9	No
	No	7005 (98.6%)	148	6767		
**Vaccination against hepatitis**	Yes	21 (0.3%)	0	21	0.49	No
	No	6994 (99.7%)	150	6844		
**History of blood transfusion**	Yes	1170 (16.7%)	30	1140	0.27	No
	No	5845 (83.3%)	120	5725		
**Dental treatment**	Yes	2630 (37.5%)	76	2554	0.0008	Yes
	No	4385 (62.5%)	74	4311		
**Surgery (minor/major)**	Yes	3309 (47.2%)	78	3231	0.2	No
	No	3706 (52.8%)	72	3634		
**History of injections**	Yes	7015 (100%)	150	6865	0	No
	No	0 (0%)	0	0		
**Sharing of comb, nail cutter, etc.**	Yes	7015 (100%)	150	6865	0	No
	No	0 (0%)	0	0		
**Visit to beauty parlor/barber shop**	Yes	7015 (100%)	150	6865	0	No
	No	0 (0%)	0	0		
**Hospitalization history**	Yes	3309 (47.2%)	85	3224	0.018	Yes
	No	3706 (52.8%)	65	3641		
**Other diseases, i.e., diabetes/T.B or any other**	Yes	656 (9.4%)	01	655	0.0002	Yes
	No	6359 (90.6%)	149	6210		
**Organ** **transplantation**	Yes	02 (0.03%)	0	02	0.8	No
	No	7013 (99.97%)	150	6863		
**Use of contaminated mourning blades**	Yes	27 (0.38%)	27	0	<0.0001	Yes
	No	6988 (99.62%)	123	6865		
**Tattooing/piercing on any part of the body**	Yes	4381 (62.5%)	123	4258	<0.0001	Yes
	No	2634 (37.5%)	27	2585		

**Table 8:** This is the reference table for knowledge assessment of the participants, which shows a significant increase in knowledge through conduction of awareness sessions and distribution of related materials.

Questions	Before Awareness		After Awareness		Chi- Square (*p* = 0.05, CI = 95% and df = 1)	Fisher’s Exact Test (*p* = 0.05 and CI = 95%)	Statistical Significance
	Yes	No	Yes	No			
Have you ever heard of a disease termed as hepatitis?	2570 (36.6%)	4445 (63.4%)	7015 (100%)	0 (0%)	0.0001	0.0001	Yes
Have you ever heard of a disease termed as hepatitis B?	2570 (36.6%)	4445 (63.4%)	7015 (100%)	0 (0%)	0.0001	0.0001	Yes
Is hepatitis B a viral disease?	2570 (36.6%)	4445 (63.4%)	7015 (100%)	0 (0%)	0.0001	0.0001	Yes
Can hepatitis B affect liver function?	1940 (27.6%)	5075 (72.4%)	7015 (100%)	0 (0%)	0.0001	0.0001	Yes
Can hepatitis B cause liver Cancer?	1207 (17.2%)	5808 (82.8%)	6200 (88.4%)	815 (11.6%)	0.0001	0.0001	Yes
Can hepatitis B affect any age group?	641 (9.1%)	6374 (90.9%)	6033 (86%)	982 (14%)	0.0001	0.0001	Yes
The early symptoms of hepatitis B are the same as cold and flu (fever, running nose, cough)	5069 (72.3%)	1946 (27.7%)	187 (2.6%)	6828 (97.4%)	0.0001	0.0001	Yes
Jaundice is one of the common symptoms of hepatitis B?	1946 (27.7%)	5069 (72.3%)	7015 (100%)	0 (0%)	0.0001	0.0001	Yes
Are nausea, vomiting, and loss of appetite common symptom of hepatitis B?	1533 (21.8%)	5482 (78.2%)	6828 (97.4%)	187 (2.6%)	0.0001	0.0001	Yes
Are there no symptoms of hepatitis B in some of the patients?	281 (4.0%)	6734 (9.6%)	5083 (72.5%)	1932 (27.5%)	0.0001	0.0001	Yes
Can hepatitis B be transmitted by un-sterilized syringes, needles, and surgical instruments?	2570 (36.6%)	4445 (63.4%)	7015 (100%)	0 (0%)	0.0001	0.0001	Yes
Can hepatitis B be transmitted by contaminated blood and blood products?	2570 (36.6%)	4445 (63.4%)	7015 (100%)	0 (0%)	0.0001	0.0001	Yes
Can hepatitis B be transmitted by using blades of the barber/ear and nose piercing?	2570 (36.6%)	4445 (63.4%)	7015 (100%)	0 (0%)	0.0001	0.0001	Yes
Can hepatitis B be transmitted by sharing of jewelry?	0 (100%)	7015 (100%)	5055 (72%)	1960 (28%)	0.0001	0.0001	Yes
Can hepatitis B be transmitted from mother to child?	2003 (28.6%)	5012 (71.4%)	7015 (100%)	0 (0%)	0.0001	0.0001	Yes
Can hepatitis B be transmitted by contaminated water/food prepared by person suffering with these infections?	4445 (63.4%)	2570 (36.6%)	0 (0%)	7015 (100%)	0.0001	0.0001	Yes
Is hepatitis B curable/treatable?	1117 (15.9%)	5898 (84.1%)	6001 (85.5%)	1014 (14.5)	0.0001	0.0001	Yes
Can hepatitis B be self-cured by body?	3568 (50.8%)	3447 (49.2%)	1101 (15.7%)	5909 (84.3%)	0.0001	0.0001	Yes
Is vaccination available for hepatitis B?	5270 (75.2%)	1745 (24.8%)	7015 (100%)	0 (0%)	0.0001	0.0001	Yes
Is a specific diet required for the treatment of hepatitis B?	4405 (52.8%)	2610 (37.2%)	6067 (86.5%)	948 (13.5%)	0.0001	0.0001	Yes

**Table 9:** This table shows the hepatitis B prevalence among different tribes of the participants.

Caste	No. of Individuals per Tribe	No. of HBsAg-Positive Individuals
Abbasi	421	10 (2.4%)
Awan	788	18 (2.3%)
Balti	105	--
Chaudhary	568	4 (0.7%)
Khawaja	936	22 (2.4%)
Mughal	688	06 (0.9%)
Pakhtoon	233	--
Peerzada	32	--
Qureshi	245	4 (1.6%)
Rajpoot	1635	21 (1.3%)
Sheikh	183	1 (0.5%)
Sudhan	310	3 (0.9%)
Syed	871	61 (7.0%)
**Total Tribes= 13**	**Total No. Individuals = 7015**	**Total HBsAg Positive = 150 (2.13%)**

**Table 10:** This table shows the results of medical history assessment of the participants.

Medical History	No. of Individuals	Male Individuals	Female Individuals
HBV Vaccination	21 (0.3%)	14 (66.6%)	7 (33.4%)
Hepatitis A	58 (0.8%)	50 (86.2%)	8 (13.8%)
Jaundice	100 (1.4%)	79 (79%)	21 (21%)
Hypocalcemia	170 (2.4%)	165 (97%)	5 (03%)
Tuberculosis	656 (9.4%)	231 (35.2%)	425 (64.8%)
Dental Surgery	2630 (37.5%)	1894 (72%)	736 (28%)
Transplantation	2 (0.03%)	1 (50%)	1 (50%)
Gastric Ulcer	41 (0.6%)	5 (12.2%)	36 (87.8%)
Gall Bladder Surgery	9 (0.2%)	3 (33.4%)	6 (66.6%)
Typhoid	438 (6.2%)	322 (73.5%)	116 (26.5%)
Chicken Pox	45 (0.6%)	31 (68.9%)	14 (31.1%)
Vision Abnormalities	85 (1.2%)	39 (45.8%)	46 (54.2%)
Renal Stones	13 (0.2%)	12 (92.3%)	1 (7.7%)
Appendicitis	162 (2.3%)	122 (75.3%)	40 (24.7%)
Any Allergy	261 (3.7%)	143 (54.8%)	118 (45.2%)
Cardiac Problems	5 (0.07%)	5 (100%)	--
Pneumonia	7 (0.09%)	2 (28.6%)	5 (71.4%)
Measles	3 (0.04%)	1 (33.4%)	2 (66.6%)

## Data Availability

Any audio and video data were not obtained during the study; however, images of the screening session are available in JPEG format, which will be provided to the journal on demand.
